# Platelet Concentration in Platelet-Rich Plasma Affects Tenocyte Behavior *In Vitro*

**DOI:** 10.1155/2014/630870

**Published:** 2014-07-23

**Authors:** Ilaria Giusti, Sandra D'Ascenzo, Annalisa Mancò, Gabriella Di Stefano, Marianna Di Francesco, Anna Rughetti, Antonella Dal Mas, Gianfranco Properzi, Vittorio Calvisi, Vincenza Dolo

**Affiliations:** ^1^Department of Life, Health and Environmental Sciences, University of L'Aquila, Italy; ^2^Postgraduate School in Orthopedics and Traumatology, University of L'Aquila, Italy; ^3^Immunotransfusion Medicine Unit, “San Salvatore” Hospital, L'Aquila, Italy; ^4^Pathological Anatomy Unit, “San Salvatore” Hospital, L'Aquila, Italy

## Abstract

Since tendon injuries and tendinopathy are a growing problem, sometimes requiring surgery, new strategies that improve conservative therapies are needed. Platelet-rich plasma (PRP) seems to be a good candidate by virtue of its high content of growth factors, most of which are involved in tendon healing. This study aimed to evaluate if different concentrations of platelets in PRP have different effects on the biological features of normal human tenocytes that are usually required during tendon healing. The different platelet concentrations tested (up to 5 × 10^6^ plt/*µ*L) stimulated differently tenocytes behavior; intermediate concentrations (0.5 × 10^6^, 1 × 10^6^ plt/*µ*L) strongly induced all tested processes (proliferation, migration, collagen, and MMPs production) if compared to untreated cells; on the contrary, the highest concentration had inhibitory effects on proliferation and strongly reduced migration abilities and overall collagen production but, at the same time, induced increasing MMP production, which could be counterproductive because excessive proteolysis could impair tendon mechanical stability. Thus, these *in vitro* data strongly suggest the need for a compromise between extremely high and low platelet concentrations to obtain an optimal global effect when inducing *in vivo* tendon healing.

## 1. Introduction

Platelet-rich plasma (PRP) is a portion of the plasma fraction that has a platelet concentration above baseline values (whole blood) [1]. PRP is obtained by centrifugation of whole blood, which separates the various components of blood according to their specific weight, and its activation through an activator, for example, thrombin, results in the formation of platelet gel (PG) [2]. Activation of platelets in PRP induces the release of several growth factors that are stored in *α*-granules, such as platelet-derived growth factor (PDGF), transforming growth factor- (TGF-) *β*, insulin-like growth factor (IGF), vascular endothelial growth factor (VEGF), fibroblast growth factor (FGF), and epidermal growth factor (EGF) [3].

The release of growth factors at the site of injury in higher concentrations than those found in whole blood [4, 5] helps the healing of tissues because these factors contribute to several required processes, such as cell proliferation, chemotaxis, cell differentiation, and angiogenesis [3, 6]. In normal healing processes, activated platelets are trapped in a clot and release approximately 95% of the growth factors, stored in a presynthesized form in *α*-granules, within the first hour. Platelets then secrete additional growth factors for another 7 days, which sustains the healing process for longer time [7]. These growth factors bind to receptors on cell membranes and activate the pathways involved in tissue healing. Therefore, PG increases platelet numbers and, consequently, the concentration of growth factors at lesion sites to induce faster healing.

Even if the concentration of most of these growth factors is higher in PRP than in respective serum, this is not true for IGF, whose level is not elevated [8–12]. This circumstance is very meaningful if we considered that, even if usually the concentration and availability of circulating IGF vary greatly among individuals and are dependent on several factors (including sex, age, genetic influences, hormones, nutritional status, and catabolic stressors) [13], there are some indications that elevated IGF level could be in some way related to cancer development [11]; IGF level in PRP, as already said, is not significantly higher than circulating level and there is no significative systemic increase of IGF after PRP administration without, consequently, an increased risk of cancer [11]. The safeness of PRP use is further highlighted if we considered that PRP is mainly an autologous product and, consequently, an excellent treatment from a safety point of view. Moreover, due to its low cost, availability, and safety, PRP has become an interesting clinical tool as a source of growth factors and is, therefore, used in a wide range of surgery fields such as oral, periodontal, maxillofacial, cosmetic, general, and orthopedic [6, 14, 15]. Clinical applications range from the treatment of nonhealing ulcers, chronic tendinopathy, and ligamentous and acute muscle injuries and as an adjuvant in bone grafting to intraoperative use in the reconstruction of anterior cruciate ligaments and in joint arthroplasty or in the repair of rotator cuffs, cartilage lesions, or tendons [6, 15].

Recently, interest in the use of PRP for tendon healing has increased because growth factors that are released from platelets could be involved in tendon-repair processes. Tendon healing occurs through inflammation, proliferation, and remodeling phases that are regulated by several growth factors, some of which are released from platelets [16]. TGF-*β* and IGF seem to be involved in fibroblast proliferation and migration, and in the following increase in collagen synthesis; VEGF has an important role in angiogenesis induction; and PDGF is involved in tissue remodeling and helps to stimulate the production of other growth factors [3, 17, 18]. To better understand the effect of PRP on human tendon cells, controlled clinical trials are important, but detailed* in vitro* studies are also critical. In most of the studies that have been performed, the concentration of PRP was expressed as a percentage, making it impossible to understand how many platelets/*μ*L were effectively used [17, 19].

The aim of this study was to fully characterize the effect of several specific concentrations of platelets (expressed as plt/*μ*L) from activated PRP (i.e., PG) on the cellular parameters of human tenocytes and on the expression of matrix metalloproteinases (MMPs) and collagen, which are usually used as markers of tendon cell biology [19].

## 2. Materials and Methods

### 2.1. Isolation and Culture of Human Tenocytes

Human tendon samples were obtained during surgical reconstruction of the anterior cruciate ligament with gracilis and/or semitendinosus tendon autograft; informed consent was obtained from all patients (Table 1). All samples used for the study were considered surgical waste and would otherwise have been discarded. The tendon samples were cleaned of the surrounding adipose tissue and peritendineum, minced, and transferred to 6-well plates containing complete medium: DMEM/Ham's F-12 culture medium (Euroclone SpA, Milan, Italy, and Sigma, St. Louis, MO, USA, resp.; 1 : 1 vol/vol) that was supplemented with 10% fetal bovine serum (FBS) (Euroclone SpA, Milan, Italy), 1 × penicillin/streptomycin (Euroclone SpA, Milan, Italy), 2 mM glutamine (Euroclone SpA, Milan, Italy), 50 *μ*g/mL gentamicin (Sigma, St. Louis, MO, USA), and 2.5 *μ*g/mL amphotericin B (Sigma, St. Louis, MO, USA). Tenocytes migrated out of the minced tissue and adhered to the bottom of the well. The cells were then successively cultured in the same medium, maintained at 37°C in a humidified atmosphere with 5% CO_2_, and trypsinized at subconfluency. The cells were used until the fourth passage.

### 2.2. Vimentin Immunostaining

Human primary gracilis and/or semitendinosus tenocytes were characterized by staining for vimentin. Briefly, cells were seeded onto glass slides and grown until they reached 80% confluency; then, they were fixed for 3 min in ice-cold methanol and 2 min in ice-cold acetone. After incubation in 10%-buffered formalin for 5 min, the slides were washed in water and stained using the EnVision FLEX, High pH system with Autostainer Link 48 (Dako, Glostrup, Denmark), which is a high-sensitivity, two-step visualization system that uses a unique, enzyme-conjugated polymer backbone that also carries secondary antibody molecules. The primary antibody that was used was the anti-vimentin mouse monoclonal antibody (ready-to-use for Autostainer Link, Clone V9, Dako, Glostrup, Denmark). After counterstaining with hematoxylin and eosin, the slides were covered with glass coverslips using an aqueous-mounting medium (Crystal/Mount, Biomeda Corporation, Foster City, CA, USA), and representative images were obtained by contrast-phase microscopy.

### 2.3. Preparation of the Platelet-Gel-Released Supernatant

Whole blood (450 mL) was collected using triple bags (Teruflex with CPD/S.A.G.M., Terumo, Rome, Italy), and each donor provided consent according to current laws (Decree Law 3, March 2005, and Law 21, October 2005, n. 219) (Table 2). Fractionation was carried out by initial centrifugation of the bag for 10 min at 22°C using a Heraeus Cryofuge 6000i centrifuge (AHSI SpA, Massa Martana (PG), Italy) at 462 g to obtain PRP and red-cell concentrates. Subsequently, the obtained PRP was subjected to a second centrifugation for 6 min at 22°C at 3932 g to produce the platelet concentrate and platelet-poor plasma. Finally, the platelets were hyperconcentrated in 10–15 mL of plasma, and PG was produced by placing tubes of platelet concentrate in a Vacutainer Plus (Becton Dickinson, Plymouth, UK) containing 5 NIH units of thrombin and adding calcium gluconate (Bioindustria Laboratorio Italiano Medicinali SpA, Novi Ligure (AL), Italy) at a 1 : 20 dilution. Subsequently the solution was allowed to clot for 5 min at 37°C, and the obtained clot was centrifuged for 10 min at 153 g to obtain a supernatant that was rich in the growth factors that had been released from the activated platelets. The supernatant was subjected to a succession of centrifugations (10 min each at 153 g, 1250 g, and 1770 g) to remove red cells, debris, and cellular stroma and was immediately used in the experimental tests. Because preliminary findings [8] showed that PG and PG-released supernatant had the same effect on cellular parameters (i.e., morphology and proliferation), all experiments were performed using PG-released supernatants, rather than GP itself, for higher feasibility in performing experiments. The initial concentration of platelets in the platelet concentrates was different in each preparation (ranging from approximately 4.5 × 10^6^ to 6 × 10^6^ plt/*μ*L); as showed in Table 2, white blood cells were also present in PRP. To obtain different concentrations of plt/*μ*L, the supernatant was diluted with medium that was supplemented with 1% FBS.

### 2.4. Proliferation Assay

Cell proliferation was determined using a 2,3-bis(2-methoxy-4-nitro-5-sulfophenyl)-2H-tetrazolium-5-carboxanilide (XTT) assay (Sigma, St. Louis, MO, USA). The metabolic reduction of XTT by living cells produces a colored, nontoxic, water-soluble formazan whose value, when measured by an ELISA reader, is directly proportional to the number of viable cells. Briefly, 1,000 cells/well were seeded onto a 96-well plate, incubated for 24 h in complete medium at 37°C and 5% CO_2_ to enable cell adhesion and spreading, starved with serum-free medium for 24 h, and then treated with the PG-released supernatant by diluting the original preparation with medium + 1% FBS to obtain different platelet concentrations (0.5–5 × 10^6^ plt/*μ*L). Tenocytes maintained in medium + 1% FBS or complete medium were used as negative and positive controls, respectively. FBS (1%) provides enough nourishment to support cell viability but does not stimulate proliferation, while complete medium is known to support cell proliferation. The cells were incubated at 37°C in a humidified atmosphere containing 5% CO_2 _for 72, 96, and 120 h. At the end of each period, an XTT assay was performed, and the OD was evaluated at 450 nm. XTT tests were performed before the positive-control cells reached confluency to prevent possible artifactual decreases in the results due to contact inhibition.

Each experiment was performed in triplicate and repeated at least twice. The data are expressed as the means ± standard deviations.

### 2.5. *In Vitro* Scratch Wound Closure Assay

The* in vitro* scratch wound closure assay was used to study directional cell migration* in vitro* and is based on the observation of cell migration into a scratch “wound” that is created on a cell monolayer. Tenocytes were cultured in 24-well microplates under normal culture conditions and allowed to reach maximum confluency. A previously sterilized, round-tipped, steel needle was used to create a wound of approximately 0.2 mm in the cellular stratum; then, the microplates were washed (to remove debris and smooth the edge of the wound) 3 times (10 min each) with medium + 1% FBS, and the cells were cultured in complete medium (positive control), medium + 1% FBS (negative control), or in PG supernatant diluted to several concentrations (0.5–3 × 10^6^ plt/*μ*L) with the same medium as the negative control. The status of the scratch wounds was monitored using phase-contrast microscopy at the beginning of the assay and at regular intervals (0, 8, 22, 30, and 46 h), and representative images were collected.

### 2.6. Gelatin Zymography

To analyze the release of MMPs from tenocytes, cells were seeded onto 6-well plates in complete medium and incubated overnight at 37°C and in 5% CO_2_ to allow for cell adhesion and spreading. When the cells were subconfluent, they were starved for 24 h and successively treated with PG supernatant diluted with medium + 1% FBS to 0.5–3 × 10^6^ plt/*μ*L for 72 h, and cells grown in medium + 1% FBS or in complete medium were used as negative and positive controls, respectively. The cells were then washed with DMEM/F-12 and incubated for 24 h in complete medium in which FBS was replaced with 0.2% LEH (Lactalbumin Enzymatic Hydrolysate, Sigma, St. Louis, MO, USA) to remove the contribution of MMPs from the serum. The recovered supernatants that contained MMPs were concentrated using Centricon Ultracel YM-10 filters (Amicon Bioseparations; Millipore Corporations, MA, USA; cutoff: 10 kDa) and analyzed via gelatin zymography. The samples were normalized by volume. Gelatin zymography was performed using sodium dodecyl sulfate-polyacrylamide gel (SDS-PAGE, 7.5%) that was copolymerized with 1 mg/mL gelatin type B (Sigma, St. Louis, MO, USA), and the supernatants were diluted in SDS-PAGE sample buffer under nonreducing conditions without heating. After electrophoresis, the gels were washed twice for 30 minutes in 2.5% Triton X-100 at room temperature and incubated overnight in a collagenase buffer (50 mM Tris-HCl, pH 7.4, containing 5 mM CaCl_2 _and 120 mM NaCl) at 37°C. The gels were then stained with Coomassie Blue R 250 (Bio-Rad, Hercules, CA, USA) dissolved in a mixture of methanol : acetic acid : water (4 : 1 : 5) for 1 h and were destained in the same solution without dye. The gelatinase activities were visualized as distinct bands that indicated proteolysis of the substrate.

### 2.7. Western Blotting

Western blotting was performed to analyze the modulation of scleraxis and the release of collagen type I from tenocytes after treatment with PG. Briefly, cells were seeded on a 6-well plate in complete medium and incubated overnight at 37°C and in 5% CO_2 _to allow for cell adhesion and spreading. When the cells were subconfluent, they were starved for 24 h and treated. For collagen analysis, the tenocytes were treated with PG supernatant that was diluted with medium + 1% FBS to 0.5−3 × 10^6^ plt/*μ*L for 72 h, and cells grown in medium + 1% FBS or in complete medium were used as negative and positive controls, respectively. The cells were then washed with DMEM/F-12 and incubated for 72 h in complete medium to allow for the release of collagen. The recovered supernatants containing collagen were concentrated using Centricon Ultracel YM-10 filters (Amicon Bioseparations, Millipore Corporations, MA, USA; cutoff: 10 kDa) and analyzed via western blotting. The samples were normalized by volume, resolved using sodium dodecyl sulfate-5% polyacrylamide gel electrophoresis (SDS-PAGE) under native, nonreducing conditions, and transferred to nitrocellulose. Nonspecific binding sites were blocked by a 1.5 h incubation with 10% nonfat dry milk in TBS-T (TBS containing 0.5% Tween-20) at room temperature. The blots were then incubated overnight with a rabbit antibody against human type I collagen (both 1*α* and 2*α* subtypes) that was diluted 1 : 1,000 (Abcam, Cambridge, UK) at 4°C, and this step was followed by incubation with a peroxidase-conjugated secondary antibody (Santa Cruz Biotechnology, Santa Cruz, CA, USA) in blocking buffer for 1 h at room temperature. After washing, the reactive bands were visualized using a chemiluminescence detection kit (SuperSignal West Femto Chemiluminescent Substrate, Thermo Scientific, Rockford, IL, USA) and the gel documentation system Alliance LD2 (Uvitec, Cambridge, UK). For scleraxis analysis, tenocytes were treated with PG supernatant that was diluted with medium + 1% FBS to 0.5−3 × 10^6^ plt/*μ*L for 18 h, and cells grown in medium + 1% FBS or in complete medium were used as negative and positive controls, respectively. Subsequently, total proteins were extracted from the tenocytes using RIPA buffer containing 50 mM Tris-HCl, pH 7.6; 150 mM NaCl; 5 mM EDTA; 1% Triton-X; 100 mM sodium fluoride (NaF); 2 mM sodium orthovanadate (Na_3_VO_4_); 2 mM sodium pyrophosphate (NaPPi); 1 mM phenylmethylsulphonyl fluoride (PMSF); and a classical protease-inhibitor cocktail (Sigma, St. Louis, MO, USA). Forty micrograms of total protein were electrophoresed by 12.5% SDS-PAGE under nonreducing, denaturing conditions and transferred to nitrocellulose membranes (Schleicher & Schuell, Dassel, Germany). Nonspecific binding sites were blocked by incubation with 10% nonfat dry milk in TBS-T containing 0.5% Tween-20 for 1.5 h at room temperature. The blot was incubated overnight with an antibody against human scleraxis (anti-scleraxis rabbit polyclonal antibody 1 : 1,000; Abcam, Cambridge, UK) at 4°, and this step was followed by an incubation with peroxidase-conjugated secondary antibody (Santa Cruz Biotechnology, Santa Cruz, CA, USA) in blocking buffer for 1 h at room temperature. After washing, reactive bands were visualized using a chemiluminescence detection kit (SuperSignal West Femto Chemiluminescent Substrate; Thermo Scientific, Rockford, IL, USA) and the gel documentation system Alliance LD2 (Uvitec, Cambridge, UK). Normalization was performed accordingly using the same protocol using a primary goat antibody that recognized actin isoforms (Santa Cruz Biotechnology, Santa Cruz, CA, USA) and a secondary rabbit anti-goat HRP-conjugated antibody (Millipore, Millipore Corporation, Billerica, MA, USA). Densitometric analysis of protein bands was performed using Alliance LD2 gel documentation system or the Image J public domain software. Relative values were calculated by comparison with negative control, defined as 1, and, where possible, normalized by the corresponding values of loading control (actin).

### 2.8. Statistical Analysis

All data shown are from at least three independent experiments and are expressed as the mean ± SD. Data were analyzed by two-way ANOVA, followed by Dunnett test, using the GraphPad Prism 4 software (GraphPad Inc., San Diego, CA, USA). Statistical significance was set at *P* < 0.05.

## 3. Results and Discussion

Tendon disorders account for a large percentage (30−50%) of all sports-related injuries and frequently lead to cessation of sport activities for long periods [20] because tendon healing is naturally slow [21]. However, tendon injuries and tendinopathy are a growing problem not only in athletes but also in elderly subjects who are still physically active [22]. Conservative treatments are not always satisfying, and they force orthopedists to resort to surgery for some patients [20]. Therefore, it is desirable to find new ways to improve conservative therapy; in this context, PRP has raised increased interest due to its high content of growth factors, most of which are involved in tendon healing [17].

Several studies on animal models showed that PRP-treated tendons healed in a shorter time and with better results regarding the quality of the tendon. In an established rat model of transected Achilles tendon, the mechanical characteristics of the tendon improved after injection of platelet concentrate [23]. In the transected tendons of rabbits, PRP seems to be able to promote the formation of scar tissue of better histological quality and improve neovascularization, which accelerates the healing process (poor vascularity seems to be one of the most important limiting factors in the healing capacity of tendons) [24]; the same effect was confirmed for surgically created, equine tendon lesions [25]. Injection of PRP into an intact rabbit patellar tendon was able to induce collagen remodeling and hypercellularity [26].

Some studies on humans have also been conducted and show an improvement in tendon healing when using PRP [27–29]. At the same time, other studies showed no significant improvement in chronic Achilles tendinopathy [20, 30, 31] or rotator cuff tendon healing [32] after treatment with PRP when compared to controls. Some authors have argued that it is difficult to correctly compare the few studies that have been performed in this field, and controlled studies are still needed to determine the real effectiveness of PRP treatment in tendon healing [33]. In addition to* in vivo* studies, some* in vitro* investigations have been performed on tenocytes isolated from several tendon origins (healthy tendons or rotator cuff tendons with degenerative tears), and these studies showed that PRP stimulated cell proliferation [3, 34–36] and collagen production [3, 35] in tenocytes. It is important to consider, however, that tenocyte biology could differ depending on donor age, anatomic origin, and status of the tendon (healthy, injured, or degenerated tendon) [35]. Most importantly, in some of these studies, the platelet concentration of PRP was unclear, and it is critical to know the exact concentration of platelets to correctly compare studies. It was clearly shown in other cell types involved in wound healing (such as endothelial cells and fibroblasts) that specific concentrations of platelets have different effects and that excessively high concentrations could be less effective [5, 8, 37]. This evidence suggests that excessively high concentrations of platelets have an inhibitory effect on the wound-healing processes and are therefore counterproductive.

For these reasons, our purpose was to evaluate the* in vitro* effect of different concentrations of platelets (and of different growth factor concentrations) on the biological features of tenocytes. As the platelet source used was platelet gel, the activated form of PRP that was obtained adding thrombin and calcium gluconate to the latter; more specifically, supernatant released from PG was used because it had already been shown that it had the same effect as PG on cellular parameters [8]. Therefore, for convenience, all experiments were performed using PG-released supernatants. Platelet concentration in the PG-released supernatants was expressed as plt/*μ*L because the concentration of the factors that are released from the platelets is assumed to be proportional to the initial concentration of plt/*μ*L, and it was assumed that the supernatants would maintain the same concentration of plt/*μ*L even if platelets were no longer present in the releasate.

The present study evaluated the ability of PG-released supernatants to affect tenocyte activities that are required for the tendon-healing process that occurs in three overlapping phases (inflammatory, proliferative, and remodeling phase). During these phases, in addition to other processes, the ECM is produced and remodeled from tenocytes, and the cells proliferate and migrate into wounds [38, 39]. Therefore, how different concentrations of platelets in PG-released supernatants were able to condition proliferation, migration into wounds, and the production of collagen type I and gelatinases was evaluated.

### 3.1. Tenocyte Characterization

**Figure 1:**
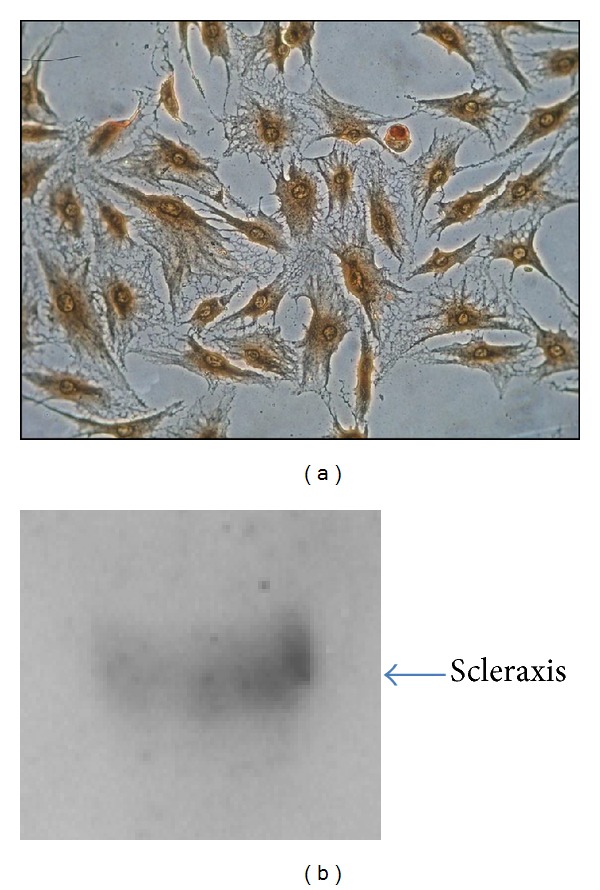
Tenocytes characterization. Tenocytes were characterized through vimentin immunostaining (a) and assay of scleraxis expression by western blot (b). Magnification 200x.

First, to confirm the identity of tenocytes, immunocytochemistry of vimentin, an intermediate filament that is characteristically found in cells of mesenchymal origin and usually used as a tenocyte marker, was performed [40, 41]. The expression of this marker was so high that counterstaining with hematoxylin and eosin was not perceptible (Figure 1(a)).

To further assess the identity of the isolated cells, the presence of scleraxis, a transcription factor that is a highly specific marker for tendons and ligaments, was evaluated [42, 43]. Western blotting confirmed the presence of this marker at a molecular weight of approximately 40 kDa (Figure 1(b)). With regard to scleraxis, we found this marker at a molecular weight of approximately 40 kDa; however, this molecule, when in its monomeric form, has a molecular weight of 22 kDa. Scleraxis belongs to a family of transcription factors (the basic helix-loop-helix [bHLH]) that are known to form heterodimers and homodimers [44, 45]; therefore, it is possible that we revealed the dimeric form of this protein. The presence of these markers, however, confirmed that the isolated cells were tenocytes.

### 3.2. Platelet-Gel-Released Supernatant Stimulates Tenocyte Proliferation

**Figure 2:**
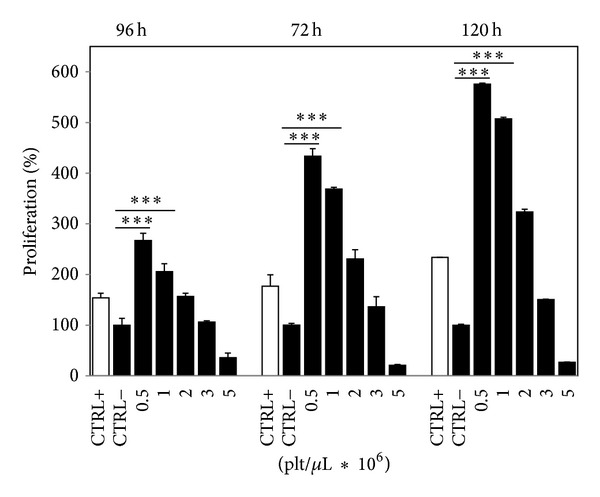
Platelet-gel-released supernatant stimulates tenocyte proliferation. The effects of different PG concentrations on tenocyte proliferation after 72, 96, and 120 h. The value obtained from untreated cells (CTRL−) was considered to be 100% proliferation. White bars (CTRL+) refer to cells grown in complete medium (positive control). Data originated in triplicate (*n* = 3) and were analyzed by two-way ANOVA, followed by Dunnett test, *** = *P* < 0.001. Error bars correspond to standard deviation.

Once the identity of the cells was confirmed, tenocyte proliferation in response to PG-released supernatant was evaluated. Because the tenocyte doubling time is approximately 96 h (data not shown), proliferation was evaluated in the treated cells at this time and 24 h after and before that time. Cells were treated with diluted PG-released supernatant to obtain the indicated concentrations (0.5 × 10^6^, 1 × 10^6^, 2 × 10^6^, 3 × 10^6^, and 5 × 10^6^ plt/*μ*L), and tests showed a dose-dependent response of the cells, with higher stimulation after 120 h (Figure 2). However, at all of the tested intervals, the optimal concentration seemed to be 0.5 × 10^6^ plt/*μ*L, which resulted in a rate of proliferation that was approximately 2.6-, 4.3-, and 5.8-fold higher than that of untreated cells after 72, 96, and 120 h, respectively. At this concentration, the proliferation rate was also higher than that of cells grown in complete medium. Higher concentrations (1.0−2.0 × 10^6^ plt/*μ*L) were able to induce proliferation, although to a lower extent, and the concentration of 3.0 × 10^6^ plt/*μ*L was ineffective after 72 h and was weakly efficient after 96 and 120 h. It was not possible to observe the trend of proliferation at longer times because, after 120 h, cells treated with the optimal concentration showed a confluence so high that it prevented further growth due to contact inhibition. As demonstrated for other cell types [5, 8, 37], high concentrations could be less effective or even counterproductive toward inducing proliferation. In fact, the highest tested concentration (5 × 10^6^ plt/*μ*L) induced cell death. For this reason, in the subsequent experiments, the highest concentration was no longer tested.

**Figure 3:**
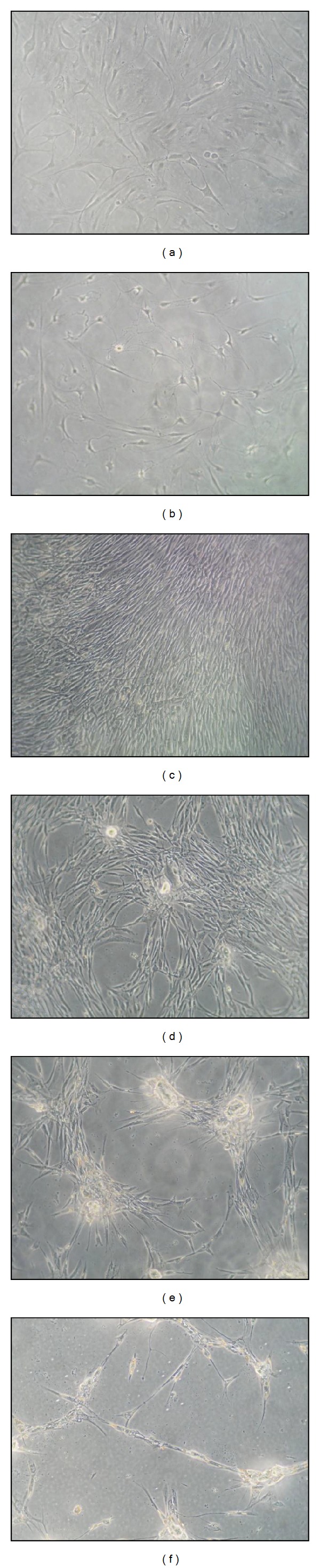
Morphological analysis of PG-supernatant-treated tenocytes. (a) Positive control (cells grown in complete medium); (b) negative control (cells grown in medium + 1% FBS); (c) cells treated with 0.5 × 10^6^ plt/*μ*L; (d) cells treated with 1 × 10^6^ plt/*μ*L; (e) cells treated with 2 × 10^6^ plt/*μ*L; and (f) cells treated with 3 × 10^6^ plt/*μ*L. Magnification 100x.

A morphological analysis of cells (Figure 3) after 120 h of treatment confirmed that, with respect to the untreated cells (Figure 3(b)), the maximum effect on human tenocyte proliferation was reached using PG at a concentration of 0.5 × 10^6^ plt/*μ*L (Figure 3(c)); the induced proliferation was even higher than that of cells grown in complete medium (Figure 3(a)). Higher concentrations induced lower stimulation and abnormal cell arrangement. These cells tended to align, cluster, and form masses (Figures 3(d)–3(f)). We are not able to explain this phenotype, but it has also been observed in fibroblasts [5]. However, this arrangement seems to be unnatural when compared to the accurate arrangement of the control cells.

### 3.3. The Effect of Platelet-Gel-Released Supernatant on Tenocyte Migration

**Figure 4:**
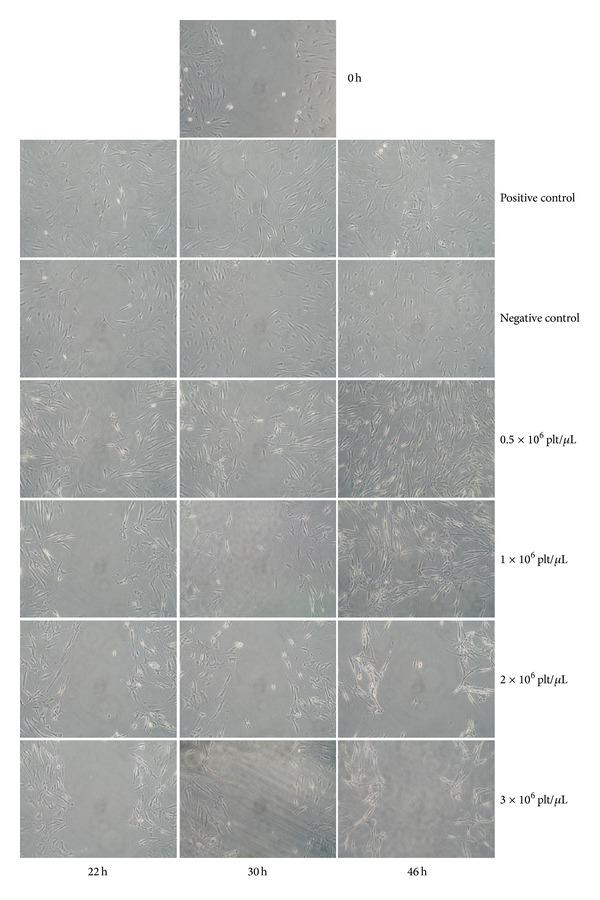
The effect of platelet-gel-released supernatant on tenocyte migration. A summary panel presenting the effects of different concentrations (rows) of platelet-gel-released supernatant on wound healing after 22, 30, and 46 h (columns). Image at 0 h is representative of the starting situation of all conditions. Magnification 100x.

The ability of the PG-released supernatant to stimulate tenocyte migration was evaluated using the scratch wound-healing assay (Figure 4).

Tenocyte migration was slow; after 8 h, no evidence of migration was observed yet (data not shown), and after 22 h the process began to be evident. Cells treated with complete medium (positive control) began to migrate into the wound as a loosely connected population after 22 h, and after 46 h the number of cells in the wound area was high. In contrast, cells treated with medium with 1% FBS (negative control) showed a decreased ability to close the wound, and after 46 h the number of cells was lower in the wound area than in the positive control. PG-released supernatant at concentrations of 0.5 × 10^6^ plt/*μ*L was not able to induce wound healing in a shorter time than the positive control, but after 46 h the number of cells that had migrated into the wound was clearly higher. Wound healing was achieved by incubation with PG-released supernatant at concentrations of 1 × 10^6^ plt/*μ*L at the same interval, but no closure of the wound occurred at higher concentrations (2 and 3 × 10^6^ plt/*μ*L). As already observed by proliferation assay (Figure 3), these concentrations induced an unusual arrangement of tenocytes. The observation of wounds was ended after 46 h because it was important that the wounds were closed by means of cell migration and not by proliferation of the cells themselves (evident effects on proliferation induced by PG treatments are significant only after 72 h, as shown in the proliferation test). For this reason, only results that were obtained within 46 h were considered significant. It seems as if PG did not influence the interval that was required for wound healing but rather the number of cells migrating into the wound, which was higher in treated than in control cells.

### 3.4. Platelet-Gel-Released Supernatant Affects the Expression of Molecules Involved in ECM Remodeling

Finally, the ability of PG to stimulate tenocyte production of gelatinases and collagen type I was evaluated. These molecules are fundamental to tendon healing because MMPs are involved in extracellular matrix remodeling, and collagen type I production is necessary to restore the extracellular matrix that is lost after injury [38, 39]. These molecules were analyzed in conditioned medium from cells that had been previously treated with PG-released supernatant. Samples for these analyses were normalized only per volume to take into account simultaneous effects on cell proliferation.

MMPs have an important role in tendon healing because, by being involved in extracellular matrix degradation, they could contribute to angiogenesis, which should improve the tendon healing process and promote the formation of scar tissue with better histological quality [24, 46, 47]. These proteases are also needed to remodel tendon injury [48]. It seems that both gelatinases, MMP-2 and MMP-9, are involved in collagen degradation, whereas collagen remodeling involves only MMP-2 [49]. In contrast, excessive proteolysis could impair the mechanical stability of tendons [3]. The expression of some MMPs that were induced in PRP-treated tendon cells has been previously investigated, although the concentration of PRP was expressed as a percentage, making it impossible to understand how many platelets/*μ*L were effectively used [3].

**Figure 5:**
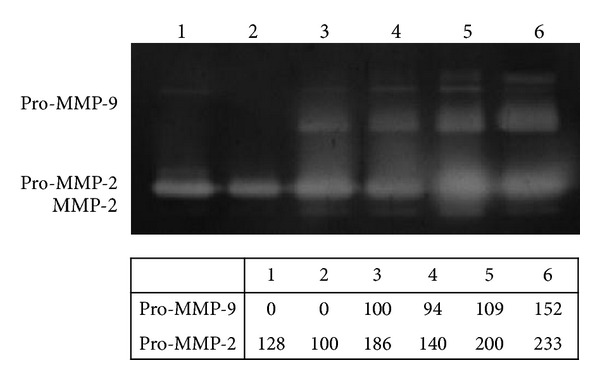
Gelatinases assay. Gelatin zymography showing the effects of different platelet-gel-released supernatant concentrations on gelatinases (MMP-2 and MMP-9) production. The figure was cropped removing upper and lower parts of gel, which contained no bands. Lane 1: positive control (cells grown in complete medium). Lane 2: negative control (cells grown in medium + 1% FBS). Lane 3: cells treated with 0.5 × 10^6^ plt/*μ*L. Lane 4: cells treated with 1 × 10^6^ plt/*μ*L. Lane 5: cells treated with 2 × 10^6^ plt/*μ*L. Lane 6: cells treated with 3 × 10^6^ plt/*μ*L. The table shows the densitometric values expressed as % volume of pro-MMP-9 and pro-MMP-2. For the pro-MMP-9 densitometric analysis the band of cells that were treated with 0.5 × 10^6^ plt/*μ*L was set at 100%, and for the pro-MMP-2 densitometric analysis the band of untreated cell (negative control) was set at 100%.

The proteolytic activity of MMPs and particularly of the gelatinases MMP-2 and MMP-9 was evaluated by analyzing supernatants from PG-treated cells through gelatin zymography. The pattern of the lytic bands is presented in Figure 5. Several bands were present in the supernatant of cells grown in complete medium: pro-MMP-2 was evident, whereas the MMP-2 band was barely visible; a weak activity at a molecular weight of approximately 120 kDa was also present, probably corresponding to gelatinase complexes (Figure 5, lane 1). In the supernatants of untreated cells, only pro-MMP-2 was present (Figure 5, lane 2), whereas in the supernatants of PG-treated cells (Figure 5, lanes 3–6) evident bands corresponding to pro-MMP-2 and activated forms of MMP-2 were observed. Lytic activity due to the pro- and active forms of MMP-2 tended to increase, with higher activity in the supernatant from cells that were treated with higher platelet concentrations (Figure 5, lane 6). In the supernatants of treated cells (Figure 5, lanes 3–6), pro-MMP-9 was also evident, but this protein was not visible in untreated or control cells (Figure 5, lanes 2 and 1, resp.); the lytic activity of pro-MMP-9 weakly increased with increased platelet concentration and high-molecular-weight bands corresponding to gelatinase complexes were also evident at a molecular weight of approximately 120 kDa (Figure 5, lanes 3–6). Densitometric analysis showed a dose-dependent trend of pro-MMP-2 and pro-MMP-9 activity (Figure 5, table).

So, our findings suggest that the bioactive molecules contained in PG were involved in inducing the production and activation of gelatinases, and we found that all tested concentrations were able to induce gelatinase activity in a dose-dependent manner. Therefore, is it possible that the highest concentrations, in addition to having a negative effect on proliferation, would have a negative effect on tendon remodeling by inducing too much lytic activity.

To further assess the ability of PG to affect extracellular matrix remodeling, we analyzed the collagen type I content in supernatants from cells treated with different concentrations of platelets. The main constituent of the tendon extracellular matrix is collagen. Collagen type I accounts for nearly 95% of the total content of the matrix, and the remaining 5% is formed from collagen types III, V, VI, XII, and XIV [50–52]. Controversial studies on the effect of PRP on collagen expression in tenocytes have been reported, and in some of those studies the expression of collagen types I and III seems to be induced [35]. Other studies have shown an increase in the total amount of collagen, even after PRP had induced a decrease in the number of collagen transcripts. The authors explain this phenomenon by stating that the collagen production per cell was reduced whilst the total collagen that was synthesized was higher due to the higher total numbers of tenocytes after cell proliferation [3, 19].

**Figure 6:**
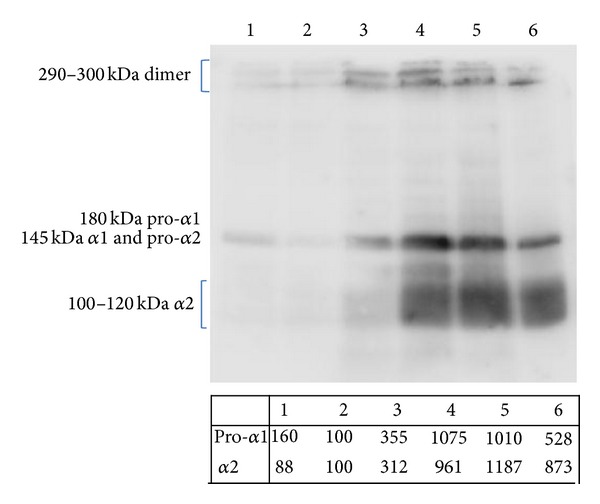
The effect of platelet-gel-released supernatant on collagen release. The effects of different PG-released supernatant concentrations on type I collagen release. Lane 1: positive control (cells grown in complete medium). Lane 2: negative control (cells grown in medium + 1% FBS). Lane 3: cells treated with 0.5 × 10^6^ plt/*μ*L. Lane 4: cells treated with 1 × 10^6^ plt/*μ*L. Lane 5: cells treated with 2 × 10^6^ plt/*μ*L. Lane 6: cells treated with 3 × 10^6^ plt/*μ*L. The table shows the densitometric values (expressed as % volume; band of negative control was set to 100%) of the pro-*α*1 and *α*2 collagen subtypes.

We analyzed the amount of collagen that was released in the cell medium after stimulation with PG-released supernatant using an antibody capable of detecting the *α*1 and *α*2 subtypes, in active, proactive, or dimer form [53]. As in previous experiments, the cell medium was normalized per volume but not per cell number to take into account the simultaneous effect on cell proliferation. As Figure 6 shows, the antibody was able to detect the *α*1 and *α*2 subtypes that were in active, proactive, or dimer form (molecular weight of 290−300 kDa for the dimer, 180 kDa for pro-*α*1, 145 kDa for *α*1 and pro-*α*2, and 100–120 kDa for *α*2). The collagen level was similar in cells grown in complete medium (Figure 6, lane 1) and in untreated cells (Figure 6, lane 2), and this level weakly increased in cells treated with lower concentrations of PG (Figure 6, lane 3). However, concentrations of 1 × 10^6^ plt/*μ*L and 2 × 10^6^ plt/*μ*L induced an evident increase in all collagen type I forms (Figure 6, lanes 4-5), and the level of collagen tended to decrease when cells were treated with the highest concentration of PG (3 × 10^6^ plt/*μ*L) (Figure 6, lane 6). Densitometric analysis was performed on the sharper bands (pro-*α*1 and *α*2) and showed that cells treated with concentrations of 1 × 10^6^ plt/*μ*L and 2 × 10^6^ plt/*μ*L produced approximately 10 times more collagen than untreated cells (Figure 6, table).

So, the data showed that PG-released supernatant was able to induce collagen type I expression at all tested concentrations when compared to untreated or control cells, and a reversal of this trend only occurred at the highest concentration that was tested (3 × 10^6^ plt/*μ*L). These data indicate that PG is able to induce the production of collagen I in tenocytes, which is necessary to restore the lost extracellular matrix after injury.

**Figure 7:**
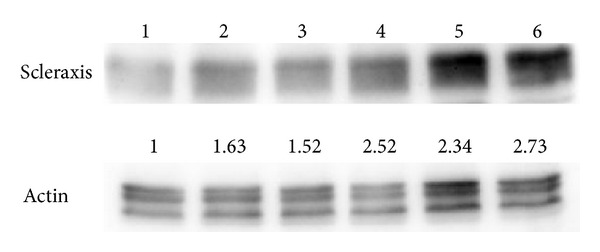
Platelet-gel-released supernatant affects scleraxis expression. The effects of different PG-released supernatant concentrations on scleraxis expression. The figures were cropped for more clarity; the removed areas contained no bands. Lane 1: negative control (cells grown in medium + 1% FBS). Lane 2: positive control (cells grown in complete medium). Lane 3: cells treated with 0.5 × 10^6^ plt/*μ*L. Lane 4: cells treated with 1 × 10^6^ plt/*μ*L. Lane 5: cells treated with 2 × 10^6^ plt/*μ*L. Lane 6: cells treated with 3 × 10^6^ plt/*μ*L. Actin detection was utilized as loading control. Values from densitometric analysis are shown on base of each protein band and were calculated as described in the Materials and Methods section.

Because it was shown that collagen expression could be regulated by scleraxis [54, 55] we aimed to understand if collagen expression induced by platelet gel was mediated by scleraxis induction. It was also shown that some stimuli, such as TGF-*β*, which is contained in platelet gel, are able to induce scleraxis expression in a time ranging from 12 to 46 h [55, 56]. Therefore, to investigate one of the molecular pathways involved in collagen stimulation, we treated tendon cells for an interval between these extremes (18 h) and successively analyzed scleraxis expression. As Figure 7 shows, treatment with increasing concentrations of platelets induced an increase in scleraxis levels, and after normalization to actin bands corresponding to cells treated with higher concentrations of platelets (Figure 7, lanes 4–6) showed higher expression of scleraxis (approximately 2.5×) than in untreated cells (Figure 7, lane 1).

These data seemed to be in contrast with previous data showing that collagen levels increased when exposed to up to 2 × 10^6^ plt/*μ*L and then decreased when exposed to 3 × 10^6^ plt/*μ*L because, in our case, scleraxis was higher in cells treated with 3 × 10^6^ plt/*μ*L. In our opinion, the reversal of this trend in collagen expression at higher concentrations is due to the simultaneous effect of the platelet gel on tenocyte proliferation. It is most likely that the collagen production per cell is increased whilst the total collagen that is synthesized is lower due to the lower proliferation of tenocytes.

## 4. Conclusions

The present study provides scientific indications of the real ability of PG to induce,* in vitro,* all the necessary mechanisms that are required for tenocytes to restore normal tissue during tendon healing* in vivo*. We are not aware of other studies of this type that were conducted on healthy tenocytes (i.e., tenocyte biology could differ depending on the status of the tendon: healthy, injured, or degenerated) [35]. Overall, our findings suggest that different concentrations of plt/*μ*L (most likely different concentrations of the growth factors that are released from the platelets) exhibit different levels of efficacy in inducing these processes. Excessively high concentrations of PG have an inhibitory effect on proliferation (and massive cell death occurs at the highest concentration that was tested), migration, and the production of collagen type I. In contrast, MMP production increased with increasing concentration until the highest concentration, which could be counterproductive because excessive proteolysis could impair tendon mechanical stability. In light of this, it is obvious that the “more is better theory” is not valid in this case because not all concentrations were equally useful. In fact, excessively high values of platelets/*μ*L seemed to be counterproductive for tenocyte biology. Therefore, we can suppose that PG can be successfully employed to facilitate human tendon regeneration due to its ability to induce cellular processes useful to tendon healing (proliferation, migration, and collagen production) and that its use could influence the strategies that are classically used in tendon reconstructive/corrective surgery. We have to be aware, anyway, that in tendon disorders tenocytes biology could be someway different from that of healthy cells used in this study, so the application of GP in degenerated tendons needs further investigation.

## Figures and Tables

**Table 1:** Sex and age of cells' donors.

Sample	Donor sex	Donor age
A	Male	53
B	Male	39
C	Female	34
D	Male	25
E	Male	37

**Table 2:** Properties of PRP.

PRP	plt/*µ*L	WBC/*µ*L	Donor sex	Donor age
A	5229000	17010	Male	52
B	4341000	8100	Female	30
C	5886000	24000	Male	50

## Abbreviations

bHLH: Basic helix-loop-helix
ECM: Extracellular matrix
EGF: Epidermal growth factor
FBS: Fetal bovine serum
FGF: Fibroblast growth factor
IGF: Insulin-like growth factor
LEH: Lactalbumin enzymatic hydrolysate
MMPs: Matrix metalloproteinases
PDGF: Platelet-derived growth factor
PG: Platelet gel
PRP: Platelet-rich plasma
SDS-PAGE: Sodium dodecyl sulfate-polyacrylamide gel electrophoresis
TGF-*β*: Transforming growth factor-*β*
VEGF: Vascular endothelial growth factor.
